# Validation of muscle oxygenation kinetics to predict aerobic fitness and exercise transition thresholds

**DOI:** 10.1113/EP092908

**Published:** 2025-08-22

**Authors:** Heru Syarli Lesmana, Ben Schroeder, Kyohei Marume, Patrick Rodrigues, Justin S. Lawley

**Affiliations:** ^1^ Department of Sport Science University of Innsbruck Innsbruck Austria; ^2^ Department of Sport Coaching Universitas Negeri Padang Padang Indonesia; ^3^ Department of Cardiovascular National Cerebral and Cardiovascular Center Suita Japan; ^4^ Division of Health, School of Sport and Human Movement University of Waikato Hamilton New Zealand

**Keywords:** aerobic fitness, near‐infrared spectroscopy, reactive hyperaemia, recovery post‐exercise

## Abstract

The aim of this study was to validate previously developed equations to predict maximal oxygen uptake (${\dot{V}}_{O_{2}\max}$) from near‐infrared spectroscopy (NIRS) during and after a period of limb ischaemia. Moreover, NIRS recovery kinetics after steady‐state exercise (SSE) could be used to monitor ${\dot{V}}_{O_{2}\max}$ and exercise intensity thresholds. Seventeen healthy adults completed a 3 min 300 mmHg pressure cuff occlusion to measure the occlusion slope, relative rate of muscle reoxygenation at 10 s (Rep 10s), baseline (*R*_bl_), peak (*R*_peak_) and area under the curve (AUC_2min_). SSE was performed at 100 W (SSE1) and 150 W (SSE2) to determine the relative rate of muscle reoxygenation (*R*1_bl_ and *R*2_bl_). Thereafter, incremental maximal cycling was used to determine ${\dot{V}}_{O_{2}\max}$, ventilatory thresholds (VTs) and gross efficiencies (GEs). The values of Rep 10s (*r* = 0.61, *p* = 0.02), *R*_bl_ (*r* = 0.53, *p* = 0.04), *R*_peak_ (*r* = 0.60, *p* = 0.02), AUC_2min_ (*r* = 0.67, *p *< 0.01) and occlusion slope (*r* = −0.68, *p* = 0.005) were correlated with absolute ${\dot{V}}_{O_{2}\max}$. After steady‐state cycling, SSE1 *R*_bl_ was correlated with absolute ${\dot{V}}_{O_{2}\max}$ (*r* = 0.67, *p* = 0.01) and relative ${\dot{V}}_{O_{2}\max}$ (*r* = 0.60, *p* = 0.02), in addition to absolute VT1 (*r* = 0.66, *p* = 0.01) and relative VT1 (*r* = 0.61 *p* = 0.02). The SSE2 *R*_bl_ was correlated with absolute ${\dot{V}}_{O_{2}\max}$ (*r* = 0.58, *p* = 0.02), relative ${\dot{V}}_{O_{2}\max}$ (*r* = 0.63, *p* = 0.02), absolute VT2 (*r* = 0.56, *p* = 0.03), relative VT2 (*r* = 0.62, *p* = 0.01) and GE2 (*r* = 0.56, *p* = 0.03). Using previously defined prediction equations, ${\dot{V}}_{O_{2}\max}$ could be predicted with a modest degree of typical error (Rep 10s, 521 mL min^−1^; *R*_peak_, 525 mL min^−1^; slope, 393 mL min^−1^). NIRS kinetic profiles during or after a period of ischaemia or after SSE are related to ${\dot{V}}_{O_{2}\max}$ and exercise intensity thresholds. Nonetheless, their predictive validity is limited to a broad estimate of the aerobic fitness of an individual.

## Abstract (highlights)

- **What is the central question of this study?**
  We aimed to validate previously developed equations to predict maximal oxygen uptake from near‐infrared spectroscopy during and after limb ischaemia. Furthermore, we investigated muscle oxygenation recovery kinetics post‐exercise to determine VO2max and ventilarory threshold.
- **What is the main finding and its importance?**
  Muscle oxygenation kinetic profiles during or after ischaemia had modest predictive validity, with the occlusion slope displaying the least source of error. The reperfusion rate of muscle oxygenation recovery kinetics after 3 min of steady‐state exercise, both low and moderate intensity, was associated with maximal oxygen uptake. Muscle oxygenation recovery kinetics after low steady‐state exercise are a good determinant of the first ventilatory threshold, and after moderate steady‐state exercise a good predictor for the second ventilatory threshold.

## 1 INTRODUCTION

Aerobic fitness is commonly estimated using various physiological parameters, including maximal oxygen uptake (${\dot{V}}_{O_{2}\max}$), the fractional utilization of ${\dot{V}}_{O_{2}\max}$ (%${\dot{V}}_{O_{2}\max}$), ventilatory thresholds (VTs) and gross energy cost (Bassett & Howley, 2000; Heigerud, 1994). These metrics are widely used to evaluate the effects of training interventions and to guide individualized exercise training intensity prescriptions. Aerobic fitness testing is relevant not only for high‐performance athletes but also for the general population, because low aerobic fitness is associated with an increased risk of cardiovascular disease and all‐cause mortality (Myers, 2003).

Although maximal incremental tests are widely used in elite sports, they have several drawbacks that include inducing fatigue and being time‐consuming for athletes with demanding training and competition schedules (Jones & Doust, 2020; Sierra, 2015). Additionally, exercise testing requires specialized, often expensive equipment that is typically available only in dedicated laboratories. From a clinical perspective, safe, non‐exhaustive testing methods would be advantageous for patients with cardiorespiratory diseases or other pre‐existing medical conditions, because many individuals cannot tolerate maximal exercise (Akalan et al., 2004; Lee & Zhang, 2021; Zugck et al., 2000). Given these limitations, alternative testing procedures have been developed to estimate ${\dot{V}}_{O_{2}\max}$ and VT outside laboratory settings using more accessible tools.

Previous studies estimating ${\dot{V}}_{O_{2}\max}$ and transition thresholds were based on predictive equations but still required maximal efforts (Buttar et al., 2019; da Silva et al., 2021; Ferreira et al., 2015; Heuberger et al., 2018). A potential simplified alternative to aerobic fitness tests is the use of near‐infrared spectroscopy (NIRS) measuring the real‐time ratio of oxygenated and deoxygenated haemoglobin and myoglobin (oxy‐ and deoxy‐[HbMb], respectively), which reports a percentage value of muscle oxygen saturation ($S_{mO_{2}}$) (Arnold et al., 2024; Barstow, 2019; Hamaoka & McCully, 2019; Jöbsis, 1977). One simple and non‐invasive solution to interrogate the skeletal muscle is the well‐established technique of limb ischaemia and post ischaemic reactive hyperaemia. This procedure has previously been shown to detect differences between low‐ and high‐fitness groups and predict variables of aerobic fitness (Beever et al., 2020; Koutlas et al., 2023). Moreover, we have previously developed equations to predict ${\dot{V}}_{O_{2}\max}$ from ischaemic limb testing using ultrasound‐based measurement of muscle blood flow and NIRS kinetics (Lesmana et al., 2025 ). However, these equations require model validation, including identification of the predictive error and model bias.

During steady‐state aerobic exercise, oxidative metabolism causes a reduction in $S_{mO_{2}}$, alongside a proportional increase in vasodilatation to meet the metabolic demand (Amin, Mugele, et al., 2021; Saltin et al., 1998). On cessation of exercise, $S_{mO_{2}}$ (reoxygenation) rises rapidly (Adami et al., 2014; Ding et al., 2001; Grassi & Quaresima, 2016; Paredes‐Ruiz et al., 2020), probably in proportion to the upstream vasodilatation. Because of the tight metabolic–flow coupling, previous work has suggested that the metric of recovery time of increasing $S_{mO_{2}}$ post‐exercise might be a surrogate for oxidative metabolism (Chance et al., 1992; Ding et al., 2001; Poole & Jones, 2012) and might be used in a similar manner to the ischaemic limb test to predict aerobic capacity (Ichimura et al., 2006; Lagerwaard et al., 2021; Paredes‐Ruiz et al., 2020). The advantage of this approach is that it requires only submaximal efforts, allowing for frequent measurements during regular training sessions.

Therefore, the aim of this study was to validate previous equations developed from non‐exhaustive ischaemic limb tests to predict ${\dot{V}}_{O_{2}\max}$ (Lesmana et al., 2025) and thereafter to examine the relationships between these metrics of resting skeletal muscle oxidative kinetics and ventilatory transition thresholds. Finally, we wanted to enlarge the evidence base that submaximal postexercise NIRS recovery kinetics can potentially be used to monitor training status.

## 2 MATERIALS AND METHODS

The study was performed according to the standards set out by the *Declaration of Helsinki*, except for registration in a database and was approved by the ethics committee of the University of Innsbruck (no. 34/2018). Written informed consent was obtained from all participants following detailed verbal explanations of the experimental protocol that included information regarding all potential risks.

### 2.1 Participants

This study enrolled 17 young healthy adults, consisting of 11 males (age, 26.5 ± 1.8 years; height, 1.81 ± 5.9 m; weight, 78.3 ± 9.8 kg) and six females (age, 23.7 ± 1.8 years; height, 1.64 ± 4.3 m; weight, 59.9 ± 5.1 kg) (for the characteristics of all participants, see Table 1). All participants were healthy and free from known cardiovascular, respiratory or metabolic diseases and medications that would affect their haemodynamic responses to exercise. Individuals with acute or chronic injuries or sickness on testing days were not considered to participate. Female menstrual cycle phase was not controlled.

**TABLE 1:** Characteristics of participants.

Characteristics (*n* = 17; 6 females, 11 males)	Value
Age (years)	25.6 ± 2.3
Height (cm)	174.8 ± 9.4
Weight (kg)	71.8 ± 12.3
Body mass index (kg m^−2^)	20.5 ± 2.7
Mid‐thigh circumference (cm)	54.7 ± 5.1
Resting heart rate (beats min^−1^)	69 ± 9
Systolic blood pressure (mmHg)	121 ± 6
Diastolic blood pressure (mmHg)	78 ± 8

### 2.2 Experimental protocols and measurements

The study consisted of two visits to the laboratory with ≥24 h resting period between visits. Participants attended the laboratory having abstained from alcohol, caffeine, smoking and exercise for 12 h and were asked to consume a light meal 4 h before testing. On the first visit, after obtaining anthropometric measurements including height (in centimetres), weight (in kilograms), thigh circumference (in centimetres) and blood pressure (in millimetres of mercury), the cuff occlusion test was performed. On the second visit, participants performed two steady‐state exercise (SSE) tests and an incremental maximal exercise test.

#### 2.2.1 Cuff occlusion test

Participants were seated on a padded chair with their lower leg hanging. A thigh cuff (Hokanson CC17, USA) sized 18 cm × 108 cm was attached at the inguinal crease of the right upper thigh regardless of leg dominance. After a 2 min resting period, the thigh cuff was inflated for 3 min via a pneumatic compressor and controlled by a custom software program that inflated the cuff to a pressure of 300 mmHg. Participants were instructed to relax the right leg, and the cuff was deflated rapidly. The participant subsequently remained still, with the right leg relaxed, for 3 min (Bleeker et al., 2005).

#### 2.2.2 Steady‐state exercise and incremental maximal exercise tests

A quiet room was provided for the test, with a temperature between 20°C and 25°C. A spirometric system (Oxycon Pro; CareFusion, Hoechbach, Germany) was used to record breath‐by‐breath gas analysis continuously throughout the SSE and maximum exercise tests and was calibrated prior to each measurement. A Bluetooth chest belt (Wear Link; Polar, Kempele, Finland) was attached to monitor heart rate (HR) and transmitted to the spirometric device.

Initially, participants performed two SSE tests, consisting of 100 W (SSE1) and 150 W (SSE2) for 3 min on a cycle ergometer (Cyclus 2, Leipzig, German). At the end of each SSE test, participants were instructed to stop pedalling immediately and maintain the right leg in a relaxed neutral position, with their feet kept clipped in the pedal and the leg extended fully (straight down, 180°) for 3 min. It was instructed that the position should keep the quadriceps as ‘unloaded’ as possible to avoid even a small amount of occlusion pressure. Between the two workloads, a 3 min recovery period was given. After the 150 W SSE test, participants rested for 5 min, then began the incremental maximal exercise test to measure the individual gas exchange thresholds and the maximum rate of oxygen consumption. Participants started cycling at 100 W, with an increase of 5 W every 15 s until they reached volitional exhaustion or the minimum cadence of 85 r.p.m. could no longer be maintained.

### 2.3 Measurements

#### 2.3.1 Skeletal muscle oxygenation by near‐infrared spectroscopy

*Cuff occlusion test*: A NIRS sensor (NIRO 200, Hamamatsu Photonics, Japan) was attached below the cuff on the most prominent part of the vastus lateralis. The $S_{mO_{2}}$ (oxyhaemoglobin/total haemoglobin × 100) was recorded continuously at a sampling frequency of 6 Hz, with the method of spatial resolved spectroscopy. The NIRS has three wavelengths (775, 810 and 850 nm) and contains two detectors placed at fixed distances 4.0 and 4.5 cm from the emitting source. A ‘black‐out’ fabric and bandage were placed over the sensor to omit light artefacts and stabilize the sensor. This sensor was chosen to replicate the data collected by Lesmana et al., 2025 and validate prediction equations.

*SSE tests*: A portable NIRS sensor (Moxy, Fortiori Design LLC, USA) was placed on the most prominent part of the vastus lateralis. This NIRS sensor sent light waves (630–850 nm) from four light‐emitting diodes into the tissue beneath it and recorded the amount of returned scattered light at two detectors positioned 12.5 and 25.0 mm from the light source (Crum et al., 2017). A ‘black‐out’ fabric and bandage were also placed over the sensor. Given that one of the primary aims of this study was to assess the predictive validity of a ‘filed‐based’ metric, a portable NIRS system was chosen, which, although limited in wavelength and penetration depth, is portable and can be used during training and racing.

### 2.4 Data analysis

*Cuff occlusion test*: The NIRS device was linked to an analog‐to‐digital converter (Powerlab: ADInstruments, Oxford, UK) with a data sampling rate of 400 Hz. The data were displayed on LabChart (LabChart 8; AD Instruments) and analysed offline. Several reoxygenation parameters were calculated in order to identify optimal signal processing. The relative rate of muscle reoxygenation, that is, the time taken to reach the baseline value (*R*_bl_), and peak reoxygenation (*R*_peak_) were calculated post‐cuff occlusion. Moreover, the reoxygenation rate over 10 s after cuff deflation [i.e. reperfusion window (Rep 10s)] was calculated based on previous data (Soares et al., 2019). The increment or delta oxygenation (*I*) and time constant of muscle reoxygenation (τ) were calculated by multiplying by 0.63. Subsequently, the relative rate of muscle reoxygenation (*R*) could be calculated with the equation: *R* = *I*/τ (Azevedo et al., 2016; Ding et al., 2001; Koutlas et al., 2023). Therefore, *R*_bl_ was calculated with the value back to baseline (*I*_bl_ /τ_bl_), and *R*_peak_ was calculated when the value reached the peak (*I*_peak_/τ_peak_). During the cuff occlusion, the oxygenation slope and integral data were calculated using LabChart software. Finally, the $S_{mO_{2}}$ area under the curve (AUC_2min_) was calculated from the time of cuff release to 2 min after cuff occlusion and also for the 3 min occlusion period (AUC occlusion) using the trapezoidal rule (Rosenberry et al., 2018). Data were excluded (*n* = 2) if the NIRS signal was lost in the 3 min after cuff release.

*Steady‐state exercise tests*: The NIRS Moxy sensor collected $S_{mO_{2}}$ data, which were transferred from Excel to LabChart for further offline analysis (LabChart 8). Mean values of $S_{mO_{2}}$ were collected for the last 30 s of baseline before SSE1 and SSE2. The relative rate of muscle reoxygenation to reach the baseline value (*R*_bl_) postexercise was calculated through previously explained methods for SSE1 *R*_bl_ and SSE2 *R*_bl_. Therefore, SSE1 *R*_bl_ was calculated with the expression SSE1 *I*_bl_/SSE τ_bl_, and SSE2 *R*_bl_ was calculated with the expression SSE2 *I*_bl_/SSE2 τ_bl_. Data were excluded (*n* = 3) if the NIRS signal was lost during the exercise and post‐exercise.

#### 2.4.1 Aerobic fitness parameters

Parameters of gas exchange and HR were averaged over 15 s. Absolute ${\dot{V}}_{O_{2}\max}$ (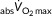) was confirmed as a plateau in oxygen uptake and recorded over the highest 30 s average. Maximal heart rate (HR_max_) and peak power output (PPO) were recorded at the corresponding time point. Relative ${\dot{V}}_{O_{2}\max}$ (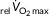) was calculated as ${\dot{V}}_{O_{2}\max}$ divided by body weight (Amin, Hansen, et al., 2021). Ventilatory thresholds (VT1 and VT2) were determined from the relationship of ${\dot{V}}_{CO_{2}}$, minute ventilation (${\dot{V}}_{E}$) and oxygen uptake (${\dot{V}}_{O_{2}}$). A disproportionate increase in ${\dot{V}}_{CO_{2}}$ and ${\dot{V}}_{E}$ relative to ${\dot{V}}_{O_{2}}$ leads to visible breakpoints representing the ventilatory gas exchange thresholds (Keir et al., 2022). Gross efficiency SS1(GE1) and SS2 (GE2) were calculated as [work accomplished (*W*)/energy expenditure (EE)]. Work accomplished (*W*) was determined as the power output during the SSE, and energy expenditure (EE) was calculated with the equation for intensities at 40%–50%${\dot{V}}_{O_{2}\max}$ (EE = 18.56${\dot{V}}_{O_{2}}$ + 2.40${\dot{V}}_{CO_{2}}$ – 4.14N) (Jeukendrup, 2005).

### 2.5 Statistical analyses

Initially, Pearson linear correlations were applied to examine the relationships between ${\dot{V}}_{O_{2}\max}$ and muscle oxygenation variables from the cuff occlusion test. The following equations were developed by Lesmana et al., 2025 to predict ${\dot{V}}_{O_{2}\max}$ from the Rep 10s (*y* = 847.0*x* + 2641), the reoxygenation rate (*y* = 795.6*x* + 2369) and the desaturation slope of $S_{mO_{2}}$ during the cuff occlusion (*y* = −18984*x* + 2272). Thus, using these equations, ${\dot{V}}_{O_{2}\max}$ was predicted from the present cuff occlusion test, and validity was evaluated as the correlation between criteria (measured ${\dot{V}}_{O_{2}\max}$) and the predicted variable (predicted ${\dot{V}}_{O_{2}\max}$), the typical error of the estimate, the coefficient of variation and the overall model bias, with data presented as Bland–Altman plots. For details on calculation methods and data presented in Bland–Altman plots, see the reliability and validity section, including freely available Excel spreadsheets, at https://sportscience.sportsci.org/ (Hopkins, 2015).

Pearson linear correlations were also applied to examine the relationships between ${\dot{V}}_{O_{2}\max}$ and gas exchange thresholds and/or metrics of efficiency and muscle reoxygenation variables from the cuff occlusion test, SSE1 and SSE2. All values are expressed as means ± SD, and α was set at *p *< 0.05. Correlation coefficients were classified following the recommendations as follows: very weak < 0.2; weak < 0.40; moderate < 0.60; strong < 0.80; and very strong > 0.80 (Evans, 1996). All statistical analyses were performed using SPSS (v.25, SPSS Inc., IBM, Chicago, IL, USA) and the graphs created in Prism (v.8.4.2, GraphPad Software Inc., La Jolla, CA, USA).

## 3 RESULTS

Mean data from the post‐occlusion test, SSE and the maximal exercise test (i.e. NIRS indices of muscle reoxygenation and aerobic fitness parameters) are presented in Tables 2, 3, 4.

**TABLE 2:** Reactive hyperaemia muscle oxygen saturation parameters.

Parameter	Value (*n* = 15)
Rep 10s (% s^−1^)	1.4 ± 0.6
*R*_bl_ (% s^−1^)	2.3 ± 1.1
*R*_peak_ (% s^−1^)	1.9 ± 0.9
*I*_bl_ (%)	16.6 ± 9.6
*I*_peak_ (%)	22.7 ± 12.9
τ_bl_ (s)	7.1 ± 2.2
τ_peak_ (s)	11.2 ± 2.8
AUC_2min_ (%)	2042 ± 993
Baseline $S_{mO_{2}}$ (%)	67.9 ± 5.5
Minimum $S_{mO_{2}}$ (%)	52.8 ± 9.8

*Note*: Values are the mean (±SD). Abbreviations: AUC_2min_, area under the curve over 2 min after cuff release; *I*_bl_, increment of muscle oxygenation back to baseline values; *I*_peak_, increment of muscle oxygenation to reach peak values; Rep 10s, reperfusion rate over 10 s; *R*_bl_, relative rate of muscle oxygenation back to baseline values; *R*_peak_, relative rate of muscle oxygenation to reach peak values; $S_{mO_{2}}$, muscle oxygen saturation; τ_bl_, time constant of muscle oxygenation to reach peak values; τ_peak_, time constant of muscle oxygenation to reach peak values.

**TABLE 3:** Steady‐state exercise parameters.

Parameter	SSE1 (*n* = 13)	SSE2 (*n* = 14)
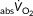 (mL min^−1^)	1715 ± 113	2196 ± 105
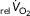 (mL kg^−1^ min^−1^)	24.2 ± 3.4	30.9 ± 4.5
HR (beats min^−1^)	127 ± 19	142 ± 20
PO (W)	100	150
RER (V˙CO2/V˙CO2V˙O2V˙O2)	0.88 ± 0.07	0.94 ± 0.08
Gross efficiency (%)	16.9 ± 1.2	19.7 ± 1.1
Relative rate of $S_{mO_{2}}$ back to bl (% s^−1^)	2.1 ± 1.4	2.9 ± 1.5
Baseline $S_{mO_{2}}$ (%)	71.4 ± 8.3	
Increment of $S_{mO_{2}}$ back to bl (%)	11.4 ± 11	10.8 ± 12.1
Time constant of $S_{mO_{2}}$ back to bl (s)	16.5 ± 11.2	23.3 ± 12
Pre‐recovery $S_{mO_{2}}$ (%)	54.3 ± 14.8	48.03 ± 17.6

*Note*: Values are the mean (±SD). Abbreviations: 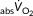, absolute volume oxygen uptake; bl, baseline; HR, heart rate; PO, power output; 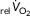, relative volume oxygen uptake; RER, respiratory exchange ratio; SSE1, steady‐state exercise 1 (100 W); SSE2, steady‐state exercise 2 (150 W); ${\dot{V}}_{CO_{2}}$, carbon dioxide output; ${\dot{V}}_{O_{2}}$, oxygen uptake.

**TABLE 4:** Incremental maximal exercise parameters.

Parameter	Value (*n* = 17)
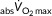 (mL min^−1^)	3773 ± 763
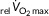 (mL kg^−1^ min^−1^)	52.7 ± 9.7
HR_max_ (beats min^−1^)	199 ± 34
PPO (W)	318 ± 60
RER (V˙CO2/V˙CO2V˙O2V˙O2)	1.18 ± 0.05
_abs_VT1 (mL min^−1^)	2771 ± 598
_rel_VT1 (mL kg^−1^ min^−1^)	38.1 ± 7.7
_abs_VT2 (mL min^−1^)	3461 ± 793
_rel_VT2 (mL kg^−1^ min^−1^)	47.5 ± 10.3

*Note*: Values are the mean (±SD). Abbreviations: 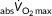, absolute maximum oxygen uptake; _abs_VT1, absolute ventilatory threshold 1; _abs_VT2, absolute ventilatory threshold 2; HR_max_, maximum heart rate; PPO, peak power output; 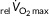, relative maximum oxygen uptake; _rel_VT1, relative ventilatory threshold 1; _rel_VT2, relative ventilatory threshold 2; RER, respiratory exchange ratio; ${\dot{V}}_{CO_{2}}$,  carbon dioxide output; ${\dot{V}}_{O_{2}}$, oxygen uptake.

### 3.1 Linear correlation between NIRS post‐occlusive reactive hyperaemia and ${\dot{V}}_{O_{2}\max}$

**FIGURE 1:**
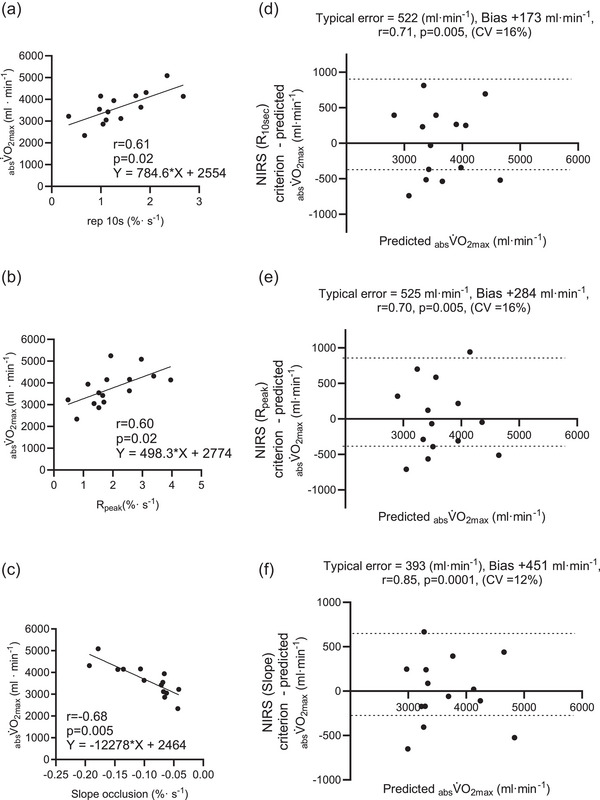
Linear correlations between absolute maximal oxygen uptake (${\dot{V}}_{O_{2}\max}$) and near‐infrared spectroscopy (NIRS)‐based metrics during and after the occlusive reactive hyperaemia (*n* = 15). (a) Reperfusion rate over 10 s (Rep 10s). (b) Relative muscle oxygenation at peak (*R*_Peak_). (c) Slope occlusion rate by NIRS from the present study. (d–f) Bland–Altman plots including validity statistics, and the dashed line donates the upper and lower confidence limits for typical error of the estimate of ${\dot{V}}_{O_{2}\max}$ based on equations developed from Lesmana et al., 2025. On the *x*‐axis, the data are the predicted absolute ${\dot{V}}_{O_{2}\max}$, and the *y*‐axis is the difference between the criterion variable (i.e. ${\dot{V}}_{O_{2}\max}$ measured with the gas analyser) and the residual as per https://sportscience.sportsci.org/2015/ValidRely.htm.

In this analysis (*n* = 15), moderate to strong positive correlations were observed between the different metrics of $S_{mO_{2}}$ after vascular occlusion and 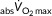. The *R*_bl_ showed a moderate correlation with 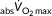 (*r* = 0.53, *p* = 0.04). The correlation with 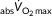 was strong for Rep 10s (*r* = 0.61, *p* = 0.02), *R*_peak_ (*r* = 0.60, *p* = 0.02) and AUC_2min_ (*r* = 0.67, *p *< 0.01). There were no correlations between reoxygenation after vascular occlusion and 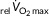 (Rep 10s, *r* = 0.42, *p* = 0.11; *R*_bl_, *r* = 0.36, *p* = 0.19; *R*_peak_, *r* = 0.41, *p* = 0.13) but AUC_2min_ showed non‐statistical significance (*r* = 0.50, *p* = 0.06) (Figure 1 and Table 5). Moreover, ischaemic stress metrics estimated during the cuff occlusion (slope, AUC and integral) were similarly correlated with 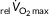 (slope, *r* = −0.51, *p* = 0.04; AUC, *r* = −0.53, *p* = 0.04; integral, *r* = −0.51, *p* = 0.05) and 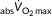 (slope, *r* = −0.68, *p* = 0.005; AUC, *r* = −0.69, *p* = 0.005; integral, *r* = −0.70, *p* = 0.002) (Figure 1 and Table 5).

**TABLE 5:** Linear correlation between maximal oxygen uptake and near‐infrared spectroscopy parameters occlusion test.

Parameter	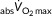 (mL min^−1^) (*n* = 15)	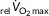 (mL kg^−1^ min^−1^) (*n* = 15)
$S_{mO_{2}}$ RH		
Rep 10s (% s^−1^)	***r* = 0.61**	*r* = 0.42
	***p* = 0.02**	*p* = 0.11
*R*_peak_ (% s^−1^)	***r* = 0.60**	*r* = 0.41
	***p* = 0.02**	*p* = 0.13
AUC_2min_ (%)	***r* = 0.67**	*r* = 0.51
	***p* = 0.006**	*p* = 0.06
*R*_bl_ (% s^−1^)	***r* = 0.53**	*r* = 0.36
	***p* = 0.04**	*p* = 0.19
Ischaemic stress		
Slope occlusion (% s^−1^)	***r* = −0.68**	***r* = −0.51**
	***p* = 0.005**	***p* = 0.04**
Integral occlusion (% s^−1^)	***r* = −0.70**	***r* = −0.51**
	***p* = 0.002**	***p* = 0.05**
AUC occlusion (%)	***r* = −0.69**	***r* = −0.53**
	***p* = 0.005**	***p* = 0.04**

*Note*: Significant relationships (*p *< 0.05) are in bold. Abbreviations: 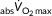, absolute maximum oxygen uptake; AUC_2min_, area under the curve over 2 min after cuff release; AUC occlusion, area under the curve 3 min occlusion period; *R*_bl_, relative rate of muscle oxygenation back to baseline values; 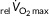, relative maximum oxygen uptake; Rep 10s, reperfusion rate over 10 s; RH, reactive hyperaemia; *R*_peak_, relative rate of muscle oxygenation to reach peak values; $S_{mO_{2}}$, muscle oxygen saturation.

### 3.2 Validation of previous prediction equations

Our previous work developed equations to predict 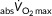 from NIRS kinetic profiles during and after ischaemic cuff occlusion. Three of the most prominent predictors were the reoxygenation rate over 10 s (Rep 10s, *y* = 847*x* +2641), peak reoxygenation (*R*_peak_, 796.5*x* + 2369) and the oxygenation slope during cuff occlusion (slope, −18984*x* + 2272) (Lesmana et al., 2025). Using these equations in the present study, 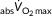 could be predicted with a modest degree of validity. All the equations had, on average, a positive bias (Rep 10s, 173; *R*_peak_, 284; slope, 451 mL min^−1^), but strong correlation coefficients (Rep 10s, 0.71; *R*_peak_, 0.70; slope, 0.85) and modest typical errors (Rep 10s, 522; *R*_peak_, 525; slope, 393 mL min^−1^).

### 3.3 Linear correlations between NIRS postexercise recovery kinetics and ${\dot{V}}_{O_{2}\max}$

**FIGURE 2:**
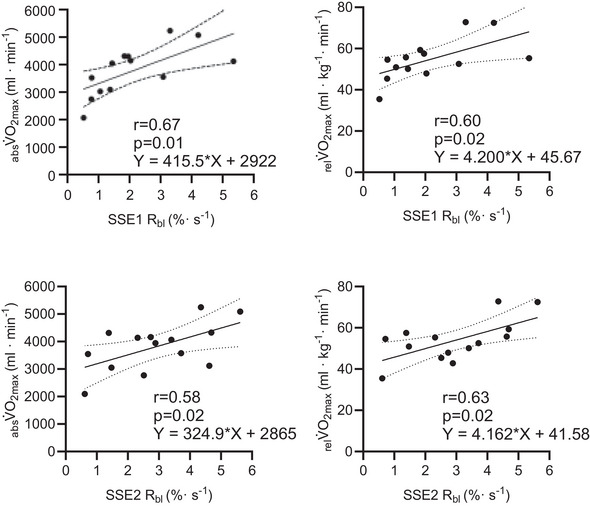
Linear correlations between absolute (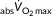) and relative (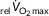) maximal oxygen uptake and metrics of postexercise recovery kinetics assessed by near‐infrared spectroscopy. Relative rate of muscle oxygenation back to baseline at post‐steady state 1 (SSE1 *R*_bl_) (*n* = 13) to 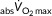 (a) and 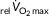 (b) back to baseline at post‐steady state 2 (SSE2 *R*_bl_) (*n* = 14) to 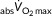 (c) and relative 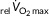 (d).

The relative rate of muscle reoxygenation after SSE showed a positive correlation with 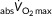 and 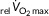. The relationship between relative muscle reoxygenation after 100 W steady‐state exercise SSE1 *R*_bl_ (*n* = 13) exhibited a strong correlation with 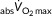 (*r* = 0.67, *p* = 0.01) and with 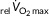 (*r* = 0.60, *p* = 0.02). The relative rate of muscle reoxygenation after SSE2 *R*_bl_ (*n* = 14) was also moderately correlated with 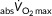 (*r* = 0.58, *p* = 0.02), and a strong correlation was observed between SSE2 *R*_bl_ and 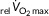 (*r* = 0.63, *p* = 0.02) (Figure 2).

### 3.4 Linear correlations between NIRS postexercise recovery kinetics and the gas exchange thresholds and metrics of efficiency

The SSE1 *R*_bl_ showed a strong positive correlation with _abs_VT1 (*r* = 0.66, *p* = 0.01) and _rel_VT1 (*r* = 0.61, *p* = 0.02), but there was no correlation between SSE1 *R*_bl_ and GE1 (*r* = 0.11, *p* = 0.70) (Table 5). The SSE2 *R*_bl_ showed a moderate positive correlation with _abs_VT2 (*r* = 0.56, *p* = 0.03), _rel_VT2 (*r* = 0.62, *p* = 0.01) and GE2 (*r* = 0.56, *p* = 0.03) (Table 6).

**TABLE 6:** Correlation matrix and regression equations between postexercise recovery kinetics and the first and second ventilatory thresholds.

	SSE1 *R*_bl_		SSE2 *R*_bl_
	*n* = 13		*n* = 14
_abs_VT1	***r *= 0.66**	_abs_VT2	***r *= 0.56**
	***p *= 0.01**		***p *= 0.03**
	***y* = 293.5*x* + 2158**		***y* = 294.5*x* + 2628**
_rel_VT1	***r *= 0.61**	_rel_VT2	***r *= 0.62**
	***p *= 0.02**		***p *= 0.01**
	***y* = 3.034*x* + 33.11**		***y* = 3.839*x* + 37.48**
GE1	*r *= 0.11	GE2	***r *= 0.56**
	*p *= 0.70		***p *= 0.03**
	*y* = 0.098*x* + 16.80		***y* = 0.383*x* + 18.52**

*Note*: Significant relationships (*p *< 0.05) are in bold. Abbreviations: _abs_VT1, absolute ventilatory threshold 1; _abs_VT2, absolute ventilatory threshold 2; GE1, gross efficiency SSE1; GE2, gross efficiency SSE2; _rel_VT1, relative ventilatory threshold 1; _rel_VT2, relative ventilatory threshold 2; SSE1 *R*_bl_, relative rate of muscle oxygenation back to baseline at steady state 1; SSE2 *R*_bl_, relative rate of muscle oxygenation back to baseline at steady state 2.

## 4 DISCUSSION

The aim of this study was to document the validity of using NIRS‐based metrics of skeletal muscle oxygenation kinetics to predict maximal aerobic capacity and exercise transition thresholds. The main findings were that $S_{mO_{2}}$ kinetic profiles during or after a period of limb ischaemia, such as the relative oxygenation recovery rate over 10 s, peak reoxygenation and the occlusion slope, had a modest predictive validity, with the occlusion slope displaying the least source of error. Moreover, postexercise NIRS recovery kinetics showed a moderate to strong relationship to ${\dot{V}}_{O_{2}\max}$ and intensity‐dependent relationships with exercise transition thresholds measured from gas exchange.

### 4.1 Prediction of maximal aerobic capacity from NIRS oxygenation kinetics during and after ischaemic limb occlusion

The ischaemic cuff test involves a brief period (∼3–5 min) of limb arterial occlusion followed by a rapid return of the circulation. Tissue hypoxia causes profound vasodilatation of the upstream circulation and, upon release of the occlusion, blood flow increases substantially (reactive hyperaemia), which causes a temporary excess of oxygen supply over metabolic demand (Behnke et al., 2009; Van Beekvelt et al., 2001). This physiological process results in a steady decline in $S_{mO_{2}}$ during cuff occlusion, because metabolic activity continues despite the cessation of oxygen delivery, and a rapid kinetic recovery of $S_{mO_{2}}$ after cuff release.

Previous research has shown that muscle deoxyhaemoglobin (HHb) and oxyhaemoglobin (O_2_Hb) changes during a single arterial occlusion test are strongly associated with aerobic fitness (${\dot{V}}_{O_{2}\max}$ and maximal aerobic velocity) (Bopp et al., 2014; Koutlas et al., 2023; Rasica et al., 2022). Moreover, our previous work (Lesmana et al., 2025) confirmed several of these relationships and developed prediction equations between muscle blood flow and a host of NIRS‐based metrics during and after a period of limb ischaemia and the ${\dot{V}}_{O_{2}\max}$ of an individual. In the present study, NIRS‐based metrics (Rep 10s, *R*_bl_, *R*_peak_, AUC_2min_ and occlusion slope) were again significantly correlated with the ${\dot{V}}_{O_{2}\max}$ of an individual. However, when using these NIRS data alongside prediction equations developed in a prior sample (Lesmana et al., 2025), model validation identified notable limitations to this approach. The primary limitation is that although the correlations between the criterion (${\dot{V}}_{O_{2}\max}$) and practical variables (NIRS metrics) are generally strong (>0.6), they are often associated with a large prediction error and 95% confidence intervals between populations used to derive the model and model validation. This is despite a very similar population in terms of cardiorespiratory fitness and identical equipment and procedures. For example, the occlusion slope had the best predictive validity, yet still had a prediction error of 393 mL min^−1^ (∼12%). Although this error includes technical error in measuring ${\dot{V}}_{O_{2}\max}$, the technical error in measuring the NIRS occlusion slope and biological variability, it is substantially greater than the between‐day variability in ${\dot{V}}_{O_{2}\max}$ measured by gas exchange (∼3%–5% with commercial systems and variable criteria for defining ${\dot{V}}_{O_{2}\max}$; Day et al., 2003; Rosdahl et al., 2010). These data highlight the limitation of previous studies that simply report correlational analyses from a single validation cohort (Bopp et al., 2014; Koutlas et al., 2023; Rasica et al., 2022). Moreover, they highlight that although NIRS kinetics might be useful to provide an estimation of the fitness of an individual in the general population, these approaches are inadequate in athletic populations or in the research setting to quantify ${\dot{V}}_{O_{2}\max}$ precisely.

### 4.2 Prediction of maximal aerobic capacity from NIRS oxygenation postexercise recovery kinetics

**FIGURE 3:**
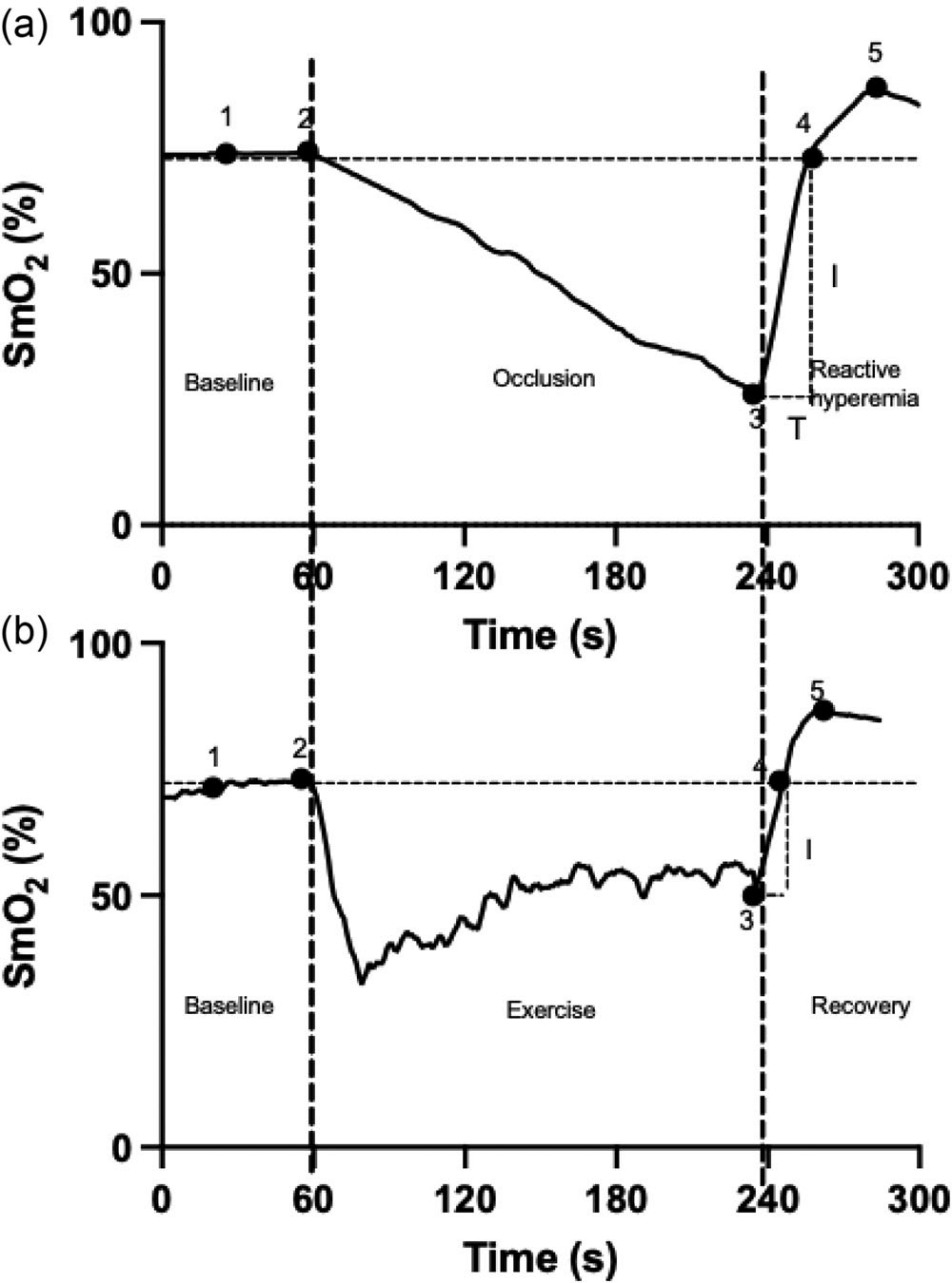
Representative changes in $S_{mO_{2}}$ during vascular occlusion test (a) and steady‐state exercise (b). Numbers: 1 = baseline; 2 = start of cuff occlusion or exercise; 3 = start of reactive hyperaemia or recovery; 4 = $S_{mO_{2}}$ back (reoxygenation) to baseline; and 5 = peak. Abbreviation: $S_{mO_{2}}$, muscle oxygen saturation.

Although the ischaemic cuff test is relatively simple to administer, repeat daily or weekly testing would be limited in most situations; thus, a simple test that could be incorporated into most training sessions would be highly advantageous. Muscle oxygenation patterns during vascular occlusion differ from those observed in SSE (Figure 3). Cuff occlusion induces local ischaemia while oxidative metabolism persists (Hayoz et al., 1995; Koutlas et al., 2023). During exercise, muscle oxygenation declines rapidly in the first few seconds because of an increased metabolic demand (Ding et al., 2001; Grassi & Quaresima, 2016; Paredes‐Ruiz et al., 2020). Owing to precise metabolic flow matching, blood flow increases (i.e. active hyperaemia) in the active skeletal muscle owing to an increase in vascular conductance, probably in proportion to the degree of tissue hypoxia. Therefore, when exercise is stopped abruptly, there is a temporary excess of oxygen supply over metabolic demand, with a rapid kinetic recovery of $S_{mO_{2}}$, similar to that observed with the cuff release.

In the present study, we observed that the rate of $S_{mO_{2}}$ recovery back to the baseline values was positively correlated with the ${\dot{V}}_{O_{2}\max}$ of an individual. These data are in line with the ischaemic cuff test, in that fitter individuals have a quicker ‘recovery’ of $S_{mO_{2}}$. Although it is tempting to ascribe these two phenomena to similar underlying physiological responses, close inspection of the data reveals a somewhat opposite interpretation. During ischaemic cuff inflation, the magnitude of ischaemic stress (i.e. ischaemic slope) is greater in fit individuals, resulting in a greater increase in vascular conductance, muscle blood flow and $S_{mO_{2}}$ recovery. During exercise, all workloads were performed at the same absolute exercise intensity (i.e. 100 and 150 W), whereby fitter individuals had a smaller reduction in $S_{mO_{2}}$. Intuitively, this means that the difference between resting and exercising $S_{mO_{2}}$ is smaller and thus the time to recover $S_{mO_{2}}$ back to the baseline value is shorter in fitter individuals.

### 4.3 Prediction of training threshold using postexercise recovery kinetics

Identifying the exercise intensity transition thresholds of an individual is a basic component of performance testing in order to identify the boundary between the moderate and heavy (first VT) and the heavy to severe (second VT) intensity domains (Poole & Jones, 2012). These thresholds are typically obtained through maximal exercise testing alongside the measurement of expired gas analysis (Iannetta et al., 2020) and are used to guide aerobic training zones (Macedo & Ide, 2025) and identify improvements attributable to training (Londeree, 1997). Because interrogating expired gas analysis aims to identify metabolic transitions at the level of the skeletal muscle, recent studies have attempted to identify exercise transition thresholds via direct measurements of skeletal muscle oxygenation profiles via NIRS. Indeed, several studies have now observed breakpoints in skeletal muscle NIRS profiles during maximal exercise tests that were correlated with the first and second gas exchange thresholds (Grassi et al., 1999; Vasquez Bonilla et al., 2023; Wang et al., 2012). As an example, Zhang et al. (2009) observed a very strong correlation (*r* = 0.97) between the second VT and the inflection point, whereby the muscle oxygen index fell rapidly during an incremental rowing exercise test. Yet these tests all require maximal exercise testing.

In the present study, we identified a moderate correlation between the relative rate of muscle reoxygenation after SSE1 and SSE2 with the first and second gas exchange threshold, respectively. These data imply that simply measuring an individual's NIRS recovery rate from short periods of SSE might shed light on their training thresholds (Belfry et al., 2012; Stöcker et al., 2016). These data are supported by more complicated NIRS‐based approaches to estimate postexercise skeletal muscle oxidative capacity with a brief period of exercise followed by repeated transient arterial occlusions (Beever et al., 2020).

It is tempting to ascribe these correlational findings from NIRS in the present study and others in the literature with quantitative measurements such as oxidative capacity, skeletal muscle capillarization or mitochondrial oxidation. Yet our previous data highlight the multicollinearity and interdependence of many of these NIRS‐based metrics alongside muscle blood flow (Lesmana et al., 2025). Moreover, NIRS recovery after SSE is likely to be the interaction of absolute ${\dot{V}}_{O_{2}}$ relative to an individual ${\dot{V}}_{O_{2}\max}$ or lactate threshold, the individual's mitochondrial function, microvascular vasodilator function and ${\dot{V}}_{O_{2}}$ offset kinetics. Thus, although in our opinion it is unlikely that these NIRS‐derived metrics encompass any one particular physiological process, it is clear from these data and others in the literature that they might be used as an integrative variable for monitoring training.

### 4.4 Limitations

One of the potential limitations of this study is that the two workloads were performed at absolute intensities (100 and 150 W) and not as a relative workload to each individual lactate threshold, critical power or ${\dot{V}}_{O_{2}\max}$. This approach has two advantages. First, the tests do not require maximal efforts in order to scale the data to absolute units. Indeed, a major aim of this work is to identify tests that avoid maximal efforts. Second, the $S_{mO_{2}}$ response during each workload is relatively a similar ${\dot{V}}_{O_{2}}$ in all individuals and thus allows for separation in terms of minimum $S_{mO_{2}}$ and $S_{mO_{2}}$ recovery patterns between different fitness levels (i.e. ${\dot{V}}_{O_{2}\max}$). Nonetheless, in athletic populations aiming to monitor their performance, scaling the initial workload prior to a training camp to the lactate threshold, critical power or a percentage of ${\dot{V}}_{O_{2}\max}$ might be optimal. On the surface, these data imply statistically that those individuals with the fastest recovery kinetics after cuff release and SSE have both the highest ${\dot{V}}_{O_{2}\max}$ and the highest VTs, which we know is not typically the case in population‐based studies (Reybrouck et al., 1986). However, again, this is likely to be an artefact of testing all participants at an absolute workload; for correct individual predictions, relative workloads might be more optimal and warrant future research.

Like previous research, our data from SSE form only the basic model development, but require a larger sample size and subsequent model validation to identify the true predictive accuracy of these recovery kinetics. Moreover, further protocol optimization and physiological justification for the NIRS kinetics are needed. For example, one anecdotal observation is that during SSE, we observed an intensity‐dependent rise in $S_{mO_{2}}$ (probably influenced by the absolute workload relative to the individual intensity transition thresholds (Grassi & Quaresima, 2016; Vasquez‐Bonilla et al., 2021), and it seems that the recovery rates are, at least in part, attributable to the degree of muscle resaturation. Development of optimization strategies to account for these factors will be important for future research and technological implementation of these strategies.

Finally, to preserve a high level of external validity, traditional scientific controls aimed at eliminating confounding factors [such as precise sensor placement (standardization of NIRS probe location), variations in the female menstrual cycle, differences in adipose or skin tissue thickness and fluctuations in muscle or cutaneous blood flow] were intentionally not applied. Although these variables are recognized as confounding in scientific experiments (Barstow, 2019; Ryan et al., 2013; Van Der Zwaard et al., 2016), they are inherent aspects of everyday life for both athletes and the general public. Therefore, for any new technology to be valuable in practice, it must demonstrate both laboratory‐based accuracy and real‐world applicability across diverse populations.

## 5 CONCLUSION

NIRS kinetic profiles during or after a period of ischaemia or after SSE are related to ${\dot{V}}_{O_{2}\max}$ and exercise intensity thresholds. Yet, their predictive validity is limited to a broad estimate of an individual's aerobic fitness. Nonetheless, these techniques seem to describe important underlying aspects of skeletal muscle physiology, and future research should aim to identify whether they are able to track changes attributable to training interventions.

## AUTHOR CONTRIBUTIONS

Heru S. Lesmana, Ben Schroeder and Justin S. Lawley were responsible for the study design and concept. Heru S. Lesmana and Ben Schroeder performed the measurements and provided data analysis. Heru S. Lesmana, Justin S. Lawley, Kyohei Marume and Patrick Rodrigues took part in interpreting data. Justin S. Lawley supervized the study. Heru S. Lesmana drafted the manuscript, and all authors reviewed and contributed to the final manuscript. All authors approved the final version of the manuscript and agree to be accountable for all aspects of the work in ensuring that questions related to the accuracy or integrity of any part of the work are appropriately investigated and resolved. All persons designated as authors qualify for authorship, and all those who qualify for authorship are listed.

## CONFLICT OF INTEREST

None declared.

## FUNDING INFORMATION

None.
